# Characterization of JAK2V617F and the JAK2 46/1 germline haplotype in myeloproliferative neoplasms in a Saudi Arabian cohort: a case-based analysis

**DOI:** 10.3389/fonc.2026.1910710

**Published:** 2026-09-15

**Authors:** Nouf Mutrib, Sana Alqarni, Abdul Ali Peer-Zada, Manar Samman, Sabiha Fatima, Sadia Arjumand, Hala Aldahshan, Khalid K. Alharbi, May M. AlRashed

**Affiliations:** 1 Department of Clinical Laboratory Sciences, College of Applied Medical Sciences, King Saud University, Riyadh, Saudi Arabia; 2 Molecular Genetics, Pathology & Clinic al Laboratory Medicine Administration, King Fahad Medical City, Riyadh, Saudi Arabia; 3 Lab Operation, Health Support Services Centre, Ministry of Health, Riyadh, Saudi Arabia

**Keywords:** JAK2 46/1 haplotype, JAK2V617F, myeloproliferative neoplasms, rs12343867, Saudi Arabia

## Abstract

**Background:**

JAK2V617F is an important mutation associated with myeloproliferative neoplasms (MPNs). The germline JAK2 46/1 haplotype is an established low-penetrance predisposition factor for MPNs across multiple ethnicities. This study aimed to characterize JAK2V617F mutation prevalence and JAK2 46/1 haplotype distribution in a Saudi Arabian MPN cohort.

**Methods:**

A retrospective cohort study was conducted at King Fahad Medical City, Riyadh, Saudi Arabia. A total of 130 participants (98 MPN patients and 32 non-MPN controls) were enrolled between January 2018 and December 2019. JAK2V617F mutation status was determined by allele-specific TaqMan qPCR. SNPs rs12343867 and rs10974900 were genotyped using validated TaqMan SNP assays to define haplotype carrier status.

**Results:**

JAK2V617F was detected in 13.1% of patients (n=17; 9 females, 8 males), with no statistically significant sex-based difference in mutation frequency. At rs12343867, the aggregate 46/1 haplotype carrier frequency was 80.7% (homozygous TT: 51.5%; heterozygous CT: 29.2%). By MPN subtype, TT homozygosity was most prevalent in ET (56.3%). By MPN subtype, the CC genotype predominated in ET (57.8%), PV (50.0%), PMF (75.0%), and non-MPN controls (54.8%).

**Conclusions:**

This is the first study to simultaneously characterize JAK2V617F and the germline JAK2 46/1 haplotype in a Saudi Arabian MPN cohort. The elevated haplotype carrier frequency supports an inherited predisposition to MPN in this population and positions the JAK2 46/1 haplotype as a candidate supplementary biomarker for risk stratification. Prospective, multi-centre studies incorporating comprehensive driver mutation profiling and population-matched healthy controls are warranted.

## 1 Introduction

Myeloproliferative neoplasms (MPNs) are a group of clonal hematopoietic stem cell disorders, including polycythemia vera (PV), essential thrombocythemia (ET), and primary myelofibrosis (PMF) (1). MPN is characterized by dysregulated hematopoietic proliferation, pro-inflammatory bone marrow microenvironment, and high risk of transformation to acute myeloid leukemia (AML) (2, 3). Although MPNs were considered acquired clonal disorders, research in the past 20 years suggests that there exist important interactions between somatic mutations and genetic factors that shape disease onset, phenotype, and progression (4). In accordance, the focus has shifted from viewing MPN as acquired conditions to recognizing them as genetically predisposed disorders.

The Janus kinase 2 (JAK2) gene (HGNC ID 6192 and chromosomal locus 9p24.1) has 142,939 base pairs and is organized into a promoter region, 25 exons, 25 introns, and a terminal regulatory region (5). Via alternative splicing, the gene generates >7 distinct transcripts, encoding three protein isoforms, A, B, and C, of the JAK2 tyrosine kinase (5, 6). JAK2 functions as a non-receptor tyrosine kinase in cytokine receptor signaling. It mediates downstream activation of the JAK-STAT pathway (7). The protein’s central role in hematopoietic cell proliferation and survival makes it a molecular target for several myeloid malignancies.

The somatic point mutation JAK2V617F (valine-to-phenylalanine substitution at position 617 in the pseudokinase domain) is the most prevalent driver mutation in MPNs. This gain-of-function mutation activates JAK2 signaling, which promotes unregulated myeloid proliferation independent of normal cytokine levels (8). JAK2V617F is detected in approximately 95% of PV cases, and in 50–60% of patients diagnosed with ET or PMF (9). Despite its diagnostic value, the V617F mutation alone does not fully account for MPN pathogenesis, as it is occasionally identified in otherwise healthy individuals (10).

The genetic make-up of MPNs broadly includes two germline categories (11). The first includes high-penetrance Mendelian syndromes affecting a single hematopoietic lineage. The second is more clinically relevant to classical MPNs; it comprises low-penetrance germline predispositions that favor the acquisition of somatic driver mutations such as JAK2V617F (11, 12). Within this framework, a germline haplotype spanning the JAK2 locus–the 46/1 haplotype, or GGCC haplotype–has become a significant inherited risk modifier for MPN development (13, 14). This is due to several non-mutually exclusive hypotheses, like enhanced baseline expression of JAK2 and neighboring genes such as INSL6 and INSL4, aberrant promoter methylation, and the presence of intronic repetitive DNA sequences that promote recombination and allele-specific overexpression (13, 15).

Despite the growing body of literature on the 46/1 haplotype in European and East Asian populations, data from Middle Eastern populations are limited. Saudi Arabia has a distinct demographic structure, consanguinity patterns, and potentially unique allelic frequencies at the JAK2 locus. Hence, the Saudi Arabians are an undercharacterized population in MPN genetics research. Understanding whether the 46/1 haplotype confers equivalent predisposition to MPNs in these patients is of both biological and clinical significance. Such data would inform population-specific risk stratification and contribute to a more globally representative understanding of MPN pathogenesis. Therefore, the objective of the present study is to investigate the association of the JAK2 46/1 haplotype with MPN susceptibility in a Saudi cohort.

## 2 Methods

### 2.1 Study design and participants

This retrospective cohort study included 130 patients visiting King Fahad Medical City (KFMC), Riyadh, Saudi Arabia, between January 2018 and December 2019. Diagnoses and subtype classification (As PV, ET, or PMF) was established in accordance with the 2016 World Health Organization (WHO) diagnostic criteria (3), using clinical, hematological, and bone marrow findings. Patients with incomplete molecular or clinical records were excluded from subtype-specific analyses as indicated.

The study adhered to the principles of the Declaration of Helsinki. Ethical approval was granted by the KFMC Institutional Review Board (IRB00008644). Written informed consent was obtained from all participants.

### 2.2 Sample collection and genomic DNA extraction

Peripheral blood samples were collected and stored in EDTA-anticoagulated tubes. The samples were processed immediately upon laboratory receipt. Genomic DNA was isolated from 200 µL of whole blood using the QIAamp DNA Mini Kit (Qiagen, Hilden, Germany; cat. no. 51304) following the manufacturer’s instructions.

Nucleic acid quality and concentration were assessed spectrophotometrically using a NanoDrop One instrument (Thermo Scientific, Waltham, MA, USA) by measuring absorbance at 260 nm, using an extinction coefficient of 50 ng•cm/µL for double-stranded DNA. Samples with an A260/A280 ratio between 1.7 and 1.9 were considered acceptable for downstream analyses. All extracted DNA was standardized to 5 ng/µL in TE buffer prior to mutation detection and genotyping assays.

### 2.3 Detection of the JAK2 V617F somatic mutation

JAK2 V617F (c.1849G>T) mutation status was determined using an allelic discrimination quantitative real-time PCR (qPCR) assay with the Ipsogen JAK2 MutaScreen Kit (Qiagen, cat. no. 673013), performed on the Rotor-Gene Q platform (Qiagen). Allelic discrimination was achieved using two TaqMan^®^ probes labelled with FAM™ (mutant allele) and VIC^®^ (wild-type allele), each incorporating a minor groove binder (MGB) at the 3′ end and a non-fluorescent quencher, enabling accurate endpoint genotyping without post-PCR processing.

During the extension phase, the perfectly complementary probe is cleaved by the 5′→3′ exonuclease activity of Taq DNA polymerase, releasing reporter fluorescence proportional to allele identity. Primer sequences are provided in **Supplementary Table 1**. Thermal cycling was conducted on the 72-tube Rotor-Gene Q rotor under the conditions detailed in **Supplementary Table 2**. Fluorescence acquisition in the FAM (Green) and VIC (Yellow) channels was performed at the endpoint of each extension step.

### 2.4 Genotyping of the JAK2 46/1 haplotype

Germline JAK2 46/1 (GGCC) haplotype status was determined by genotyping two tagging single-nucleotide polymorphisms (SNPs), rs12343867 and rs10974900 (both harbouring a C>T variant). Genotyping was performed using the Type-it Fast SNP Probe PCR Kit (Qiagen, cat. no. 206042) in conjunction with predesigned Applied Biosystems TaqMan SNP Genotyping Assays (human C:31941689_10, rs12343867 and rs10947900; cat. no. 4351379; Thermo Fisher Scientific, Waltham, MA, USA).

Reactions were assembled in Applied Biosystems MicroAmp Optical 96-well plates (Thermo Fisher Scientific), sealed, and centrifuged briefly to eliminate air bubbles before cycling. PCR amplification was performed on an Applied Biosystems 7500 Real-Time PCR System (Thermo Fisher Scientific). Hardy–Weinberg equilibrium was assessed for each SNP genotype distribution across the study population. Three genotypic categories were distinguished: homozygous C/C (non-carrier), heterozygous C/T (carrier), and homozygous T/T (carrier). Representative allele discrimination plots for each genotype class are displayed in **Figure 1**.

**Figure 1:**
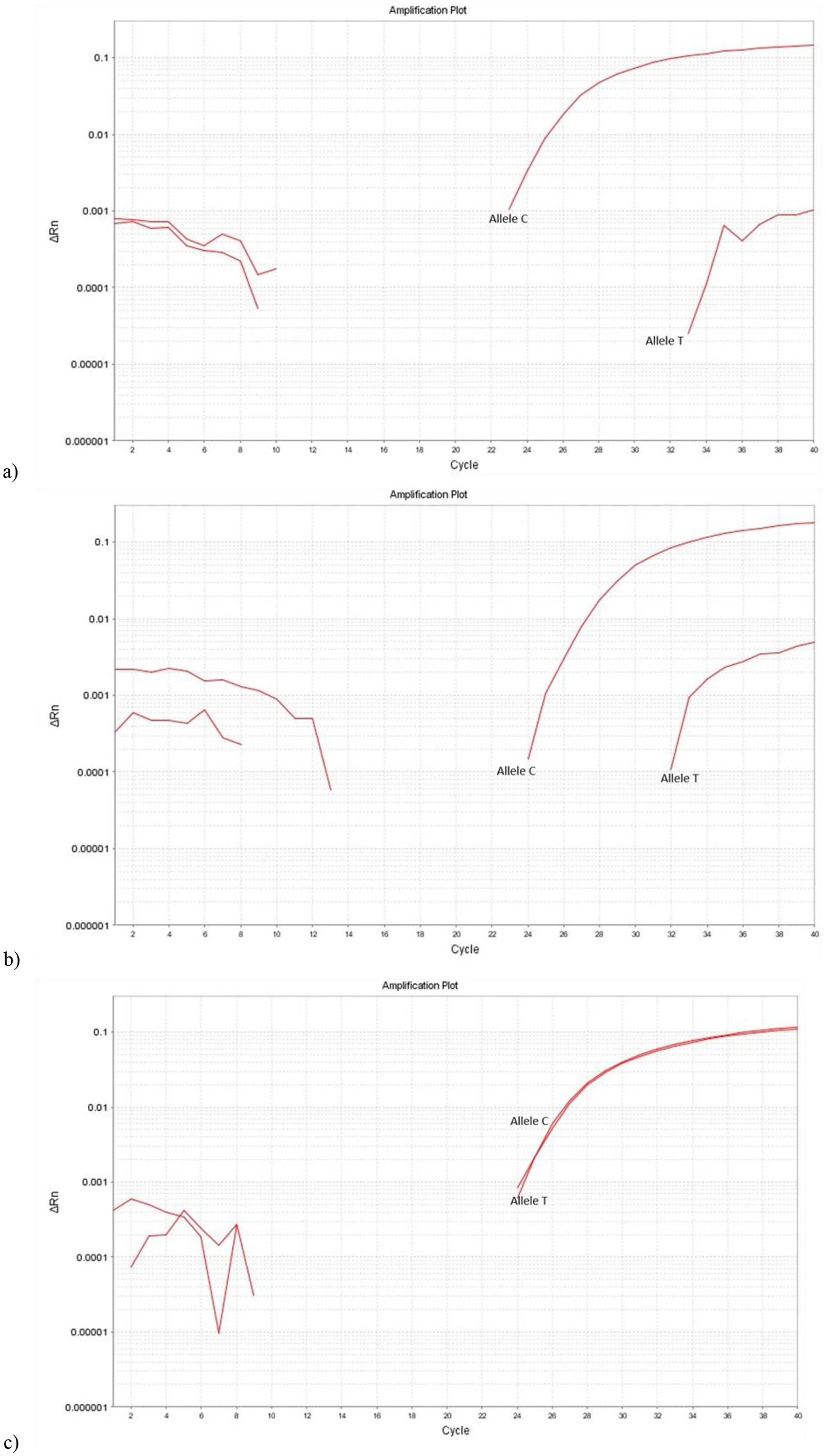
Allelic discrimination plot. **(a)** Homozygous C/C genotype at rs12343867. C-allele value: 0.171; T-allele value: 0.006. This genotype is classified as a non-carrier of the JAK2 46/1 haplotype; **(b)** Homozygous T/T genotype at rs12343867. C-allele value: 0.09; T-allele value: 0.199. Despite visual similarity to the C/C pattern, the two genotypes are distinguished by their respective allele discrimination values; **(c)** Heterozygous C/T genotype at rs12343867. C-allele value: 0.126; T-allele value: 0.125. The approximately equal allele values confirm heterozygosity.

### 2.5 JAK2 exon sequencing

Among the 130 MPN cases, 20 (15.4%) were negative for both the JAK2 V617F mutation and the 46/1 haplotype; 16 of these were selected for targeted Sanger sequencing to identify alternative predisposing variants. PCR amplification was performed across JAK2 exons 3–24, with the exception of exons 13, 14, and 15, given their established correlation with the V617F substitution. Exon-spanning primers were designed using the M13 tail strategy (forward: 5′-GTAAAACGACGGCCAGT-3′; reverse: 5′-CAGGAAACAGCTATGACC-3′) to facilitate bidirectional Sanger sequencing (see **Supplementary Table 1** for all primer sequences).

Template DNA was diluted to 20 ng/µL using the equation C_1_V_1_ = C_2_V_2_ prior to amplification. Amplification was carried out on an Applied Biosystems Veriti 96-well Thermal Cycler (Thermo Fisher Scientific). Amplicon integrity was confirmed by agarose gel electrophoresis (1.6% w/v agarose in 1× TAE buffer) with visualisation under UV illumination using a Syngene NuGenius gel documentation system (Syngene, Cambridge, UK). Amplicons were purified and submitted for bidirectional Sanger sequencing; resulting chromatograms were aligned to the human JAK2 reference sequence (NM_004972.4) using publicly available alignment software.

### 2.6 Statistical analysis

Genotype and allele frequencies were derived by direct counting. Associations between 46/1 haplotype status, JAK2 V617F mutation, and MPN subtypes were evaluated by the chi-square test or Fisher’s exact test, as appropriate for expected cell frequencies. All statistical analyses were conducted using IBM SPSS Statistics (IBM Corp., Armonk, NY, USA). A two-tailed p-value < 0.05 was considered statistically significant.

## 3 Results

### 3.1 Baseline characteristics and hematological parameters

A total of 130 blood samples were prospectively collected from patients, comprising 98 samples with confirmed MPNs and 32 control samples without MPN. The 98 MPN-positive samples were classified as 64 cases of ET (49.2%), 30 cases of PV (23.1%), and 4 cases of PMF (3.1%). All diagnoses were established in accordance with the 2016 WHO criteria, incorporating morphological, clinical, and molecular genetic assessments. The overall cohort consisted of 72 males (55.4%) and 58 females (44.6%). Age at presentation spanned from less than 10 years to 60 years or above (**Supplementary Table 3**).

Complete blood count (CBC) parameters were evaluated for each MPN subtype and compared to non-MPN control values (**Table 1**). ET patients exhibited a mean platelet count of 480.42 × 10^9^/L, substantially exceeding the upper reference limit of 435 × 10^9^/L. PV patients demonstrated a mean red blood cell (RBC) count of 5.87 × 10^12^/L and a mean haemoglobin of 16.20 g/dL (reference: 13.5–18.0 g/dL), both indicative of erythrocytosis. PMF patients exhibited the highest mean platelet count of 644.50 × 10^9^/L as well as the highest mean white blood cell (WBC) count (12.63 × 10^9^/L), both above the upper reference limits. Non-MPN controls had elevated mean WBC (17.49 × 10^9^/L).

**Table 1:** Summary of CBC-based hematological parameters stratified by MPN diagnosis and non-MPN controls.

Variable	ET (n=64)	PV (n=30)	PMF (n=4)	Non-MPN controls (n=32)
Mean WBC ×10^9^/L	8.28	11.00	12.63	17.49
(Reference: 3.9–11.0)	—	↑	↑	↑
Mean RBC ×10^12^/L	5.06	5.87	4.53	4.69
(Reference: 3.9–4.6)	↑	↑↑	Normal	Normal
Mean Haemoglobin (g/dL)	13.23	16.20	13.03	13.21
(Reference: 13.5–18.0)	↓	↑	↓	↓
Mean Platelet Count ×10^9^/L	480.42	325.07	644.50	297.94
(Reference: 155–435)	↑	Normal	↑↑	Normal

Reference ranges are indicated in parentheses. Directional arrows indicate deviation from the reference range (↑: elevated; ↑↑: markedly elevated; ↓: below reference range.

### 3.2 JAK2V617F mutation frequency

Of the 130 MPN samples analysed, 17 (13.1%) were found to be JAK2V617F-positive, while the remaining 113 (86.9%) were JAK2V617F-negative. Among JAK2V617F-positive cases, 9 were female (52.9%), and 8 were male (47.1%). Within the male sub-cohort (n=72), the mutation was detected in 8 individuals (11.1%), compared with 9 of 58 females (15.5%) in the female sub-cohort (**Table 2**). No statistically significant sex-based difference in mutation frequency was observed.

**Table 2:** Distribution of the JAK2V617F mutation by sex in the MPN cohort (n=130).

Sex	JAK2V617F positive, n (%)	JAK2V617F negative, n (%)
Male	8 (6.15)	64 (49.23)
Female	9 (6.92)	49 (37.69)
Total	17 (13.08)	113 (86.92)

### 3.3 Genotyping of the JAK2 46/1 haplotype—SNP rs12343867

Genotyping of rs12343867 was successfully completed in all 130 samples. The homozygous T/T genotype was the most prevalent, observed in 67 individuals (51.5%), followed by the heterozygous C/T genotype in 38 individuals (29.2%), and the homozygous C/C (non-carrier) genotype in 25 individuals (19.2%). When the T-allele carrier categories (homozygous T/T and heterozygous C/T) were aggregated, the overall JAK2 46/1 haplotype carrier frequency was 80.7% (n=105) in the MPN cohort.

Analysis of rs12343867 genotype distribution according to JAK2V617F mutational status revealed a notably distinct pattern in mutation-positive versus mutation-negative patients (**Figure 2a**). Among the 17 JAK2V617F-positive patients, 8 (47.1%) displayed the homozygous C/C non-carrier genotype, 7 (41.2%) were homozygous T/T, and only 2 (11.8%) were heterozygous C/T. By contrast, in the 113 JAK2V617F-negative patients, the predominant genotype was homozygous T/T (53.1%, n=60), followed by heterozygous C/T (31.9%, n=36) and homozygous C/C (15.0%, n=17). Notably, the homozygous C/C non-carrier genotype was substantially enriched among mutation-positive cases (47.1%) relative to mutation-negative cases (15.0%), suggesting a possible inverse relationship between the rs12343867 T-allele carrier status and somatic JAK2V617F acquisition in this cohort.

**Figure 2:**
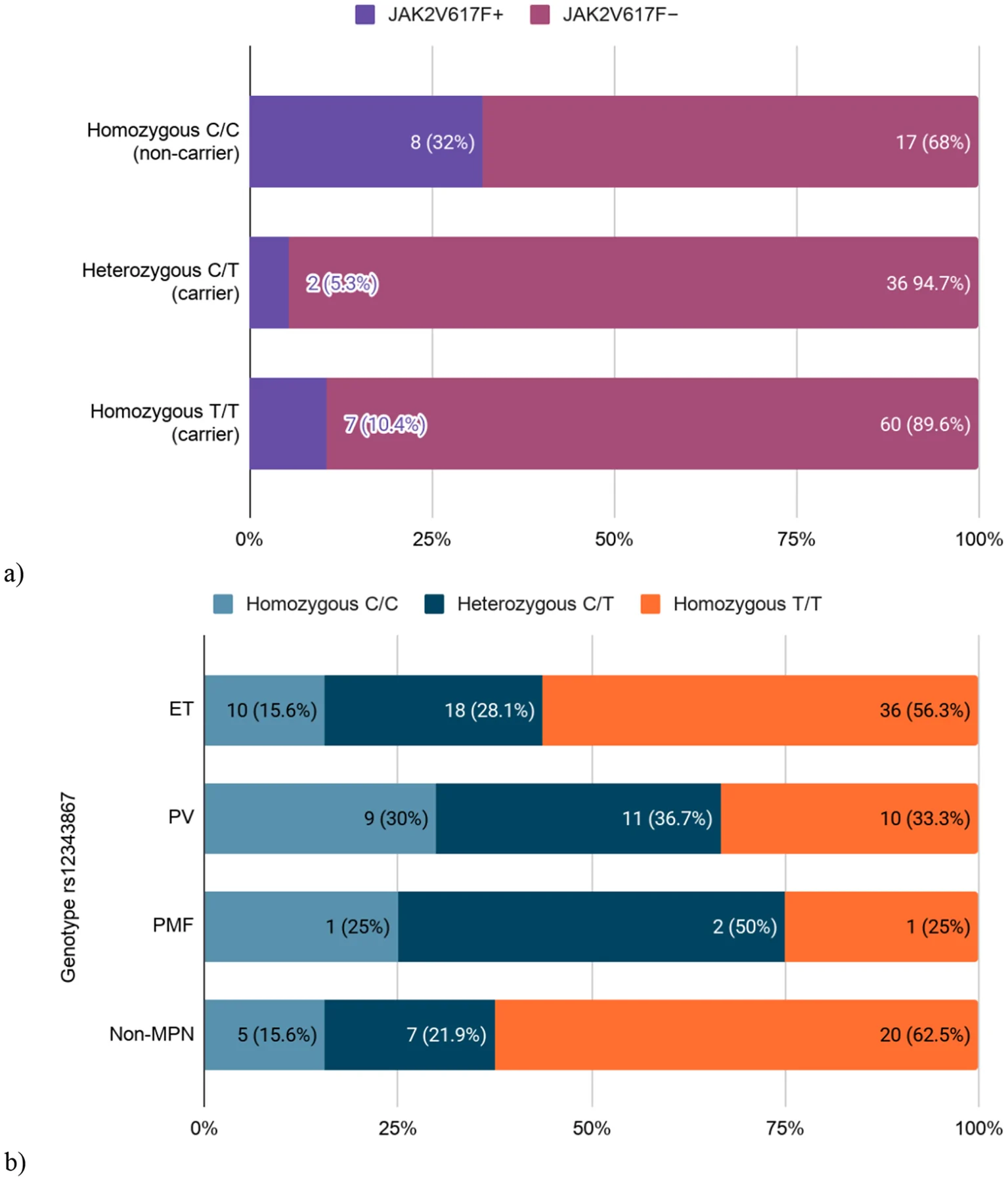
Graphs illustrating the proportion of rs12343867 genotypes based on **(a)** JAK2V617F mutation status; **(b)** MPN diagnostic category.

The distribution of rs12343867 genotypes across ET, PV, PMF, and non-MPN control groups is presented in **Figure 2b**. Among ET patients (n=64), the homozygous T/T genotype was the most common, identified in 36 cases (56.3%), followed by C/T in 18 (28.1%) and C/C in 10 (15.6%). A similar carrier-predominant pattern was observed in non-MPN controls, where 62.5% carried the homozygous T/T genotype (n=20) and 21.9% were heterozygous C/T (n=7). In the PV subgroup (n=30), the distribution was more evenly spread: C/C in 9 (30.0%), C/T in 11 (36.7%), and T/T in 10 (33.3%), indicating a higher non-carrier frequency relative to ET and non-MPN groups. In the PMF subgroup (n=4), the heterozygous C/T genotype was most prevalent (50.0%, n=2), with one case each of C/C (25.0%) and T/T (25.0%), although the small sample size limits interpretation of these proportions.

### 3.4 Genotyping of the JAK2 46/1 haplotype—SNP rs10974900

The second tagging SNP of the JAK2 46/1 haplotype, rs10974900 (C>T), was successfully genotyped in 129 of the 130 samples (99.2%); one sample yielded an indeterminate result and was excluded from this analysis. The homozygous C/C genotype was the most frequent, observed in 72 individuals (55.8%), followed by the heterozygous C/T genotype in 40 (31.0%), and the homozygous T/T genotype in 17 (13.2%). The aggregate 46/1 haplotype carrier frequency (C/T + T/T) for this SNP was 44.2% (n=57), contrasting markedly with the 80.7% carrier frequency observed for rs12343867, which reflects the different linkage disequilibrium properties of the two SNPs within the haplotype block.

The distribution of rs10974900 genotypes was markedly skewed among JAK2V617F-positive patients (**Figure 3a**). Among the 17 mutation-positive cases, 14 (82.4%) carried the homozygous C/C genotype, and 3 (17.6%) were heterozygous C/T; strikingly, no JAK2V617F-positive patient displayed the homozygous T/T genotype. In the 112 mutation-negative cases, the homozygous C/C genotype remained the most common (51.8%, n=58), but the carrier genotypes were substantially more represented: C/T in 37 cases (33.0%) and T/T in 17 cases (15.2%). The complete absence of T/T homozygosity in the JAK2V617F-positive group suggests that the T-allele of rs10974900 may be incompatible with, or strongly counter-selected against, the JAK2V617F somatic mutation in this Saudi cohort.

**Figure 3:**
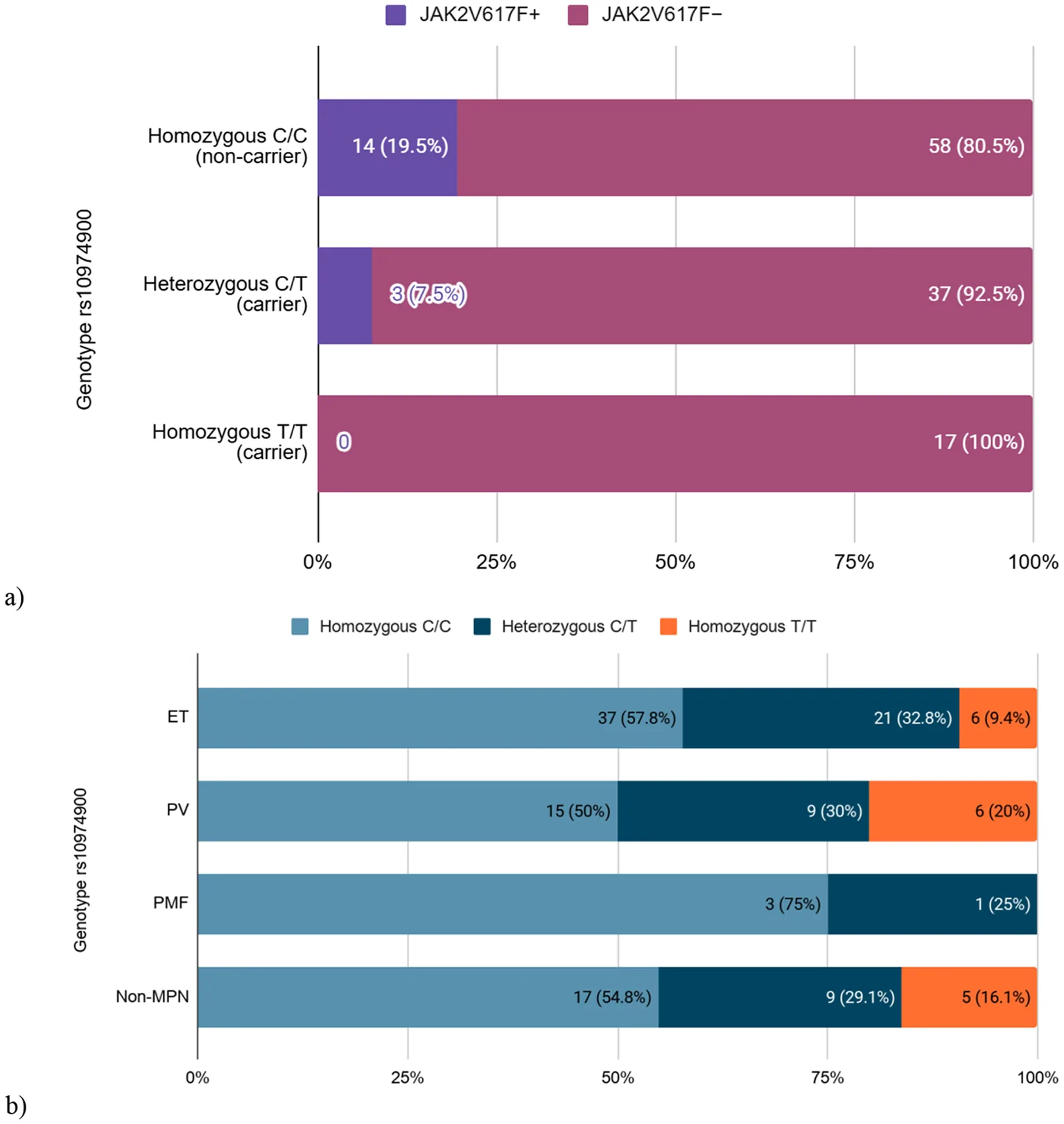
Graphs illustrating the proportion of rs10974900 genotypes based on **(a)** JAK2V617F mutation status; **(b)** MPN diagnostic category.

**Figure 3b** presents the rs10974900 genotype distribution across MPN subtypes and non-MPN controls. In ET patients (n=64), the C/C non-carrier genotype was most prevalent (57.8%, n=37), followed by C/T in 21 cases (32.8%) and T/T in 6 cases (9.4%). A similar pattern was observed in PV (C/C: 50.0%; C/T: 30.0%; T/T: 20.0%) and non-MPN controls (C/C: 54.8%; C/T: 29.0%; T/T: 16.1%). The PMF subgroup exhibited the highest non-carrier frequency (C/C: 75.0%, n=3; C/T: 25.0%, n=1), with no T/T homozygotes identified, though the very small PMF sample (n=4) precludes firm conclusions. Collectively, the predominance of the C/C genotype across all diagnostic groups suggests that the T-allele of rs10974900 is relatively uncommon in this Saudi population.

## 4 Discussion

The present retrospective cohort study investigated the prevalence of the JAK2V617F mutation and the germline JAK2 46/1 haplotype in 98 patients with MPN and 32 non-MPN controls. The JAK2V617F mutation was detected in 13.1% (n = 17) of the cohort. Analysis of rs12343867 revealed that 80.7% of patients carried the 46/1 haplotype (homozygous TT: 51.5%; heterozygous CT: 29.2%), while 19.2% were CC (nullizygous). For rs10974900, homozygous CC was the most frequent genotype (55.8%), followed by heterozygous CT (31.0%) and homozygous TT (13.2%). These observations suggest that although the JAK2V617F mutation may be a contributing factor to MPNs, it was not a predisposing factor in this study cohort. On the contrary, the germline SNPs may predispose to MPNs irrespective of the JAK2V617F status.

The JAK2V617F mutation is a somatic gain-of-function point mutation arising from a guanine-to-thymine transversion at position 1849 in exon 14 of the JAK2 gene, resulting in valine-to-phenylalanine substitution at codon 617 (5). Multiple landmark studies established the mutation frequency at approximately 95–98% in PV, 50–60% in ET, and 50–60% in PMF (16, 17). Our findings diverge from the previous studies as the JAK2V617F mutation was detected in only 13.1% of the total MPN cohort. The low mutation rate underscores the diagnostic challenge in BCR-ABL1-negative MPNs, where a significant proportion of patients, particularly those with ET and PMF, are JAK2V617F-negative and may carry alternative driver mutations in CALR or MPL (18–20). The relatively high proportion of JAK2V617F-negative cases in this cohort, therefore, likely reflects an underlying burden of CALR- and MPL-mutated disease. This molecular characterization was not carried out in this study and represents an important direction for future work.

The germline JAK2 46/1 haplotype (also termed the GGCC haplotype) is defined by four SNPs in complete linkage disequilibrium spanning introns 10 to 15 of the JAK2 gene: rs3780367, rs10974944, rs12343867, and rs1159782 (21). In 2009, three independent research groups demonstrated that this germline haplotype confers susceptibility to BCR-ABL1-negative MPN (22–24). A subsequent meta-analysis of 26 observational studies by Li et al. enrolling 8,561 cases confirmed that the 46/1 haplotype significantly elevated MPN risk (OR = 2.19, 95% CI = 1.86–2.57, P < 0.0001 for the C vs T allele at rs10974944; and OR = 3.16, 95% CI = 2.14–4.65 for CC vs TT at rs12343867) (25). The present study extends these observations to a Saudi Arabian population for the first time.

At rs12343867, the 46/1 haplotype carrier rate was 80.7% (TT homozygous: 51.5%; CT heterozygous: 29.2%) in this study population. This is substantially higher than the 24% frequency reported in healthy general populations by Anelli et al. and the 40–80% reported across JAK2V617F-positive MPN patients in European studies (8, 23). The elevated carrier frequency is noteworthy and supports the hypothesis that 46/1 haplotype predisposition is present across diverse ethnicities, consistent with data from North American, European, Japanese, and Brazilian populations (22, 26, 27). A Moroccan study similarly found a strong association between the JAK2 46/1 haplotype and MPN development, affirming that this predisposition is not restricted to Caucasian populations (28).

The observed association between the 46/1 haplotype and MPN occurrence is more consistent with the “fertile ground” hypothesis, which proposes that the inherited haplotype provides a favourable biological context for the emergence and/or clonal expansion of disease-associated mutations, including JAK2V617F (24, 29, 30). In contrast, our findings do not support the “hypermutability” hypothesis (23), whereby the 46/1 haplotype is presumed to increase the intrinsic rate of acquisition of JAK2 mutations. However, given the observational nature of this study and the absence of functional analyses, the present data cannot prove or disprove either hypothesis; it would require additional functional or mechanistic evidence.

The genotype distribution for the second SNP is different from that of rs12343867, as homozygous CC was the most common (55.8%) and homozygous TT the least frequent genotype (13.2%). No JAK2V617F-positive patients carried the TT genotype at rs10974900, while 82.4% of JAK2V617F-positive patients were CC. The enrichment of the CC genotype in JAK2V617F-positive cases may indicate linkage with genetic variants that define the JAK2 46/1 haplotype; however, formal haplotype analysis would be required to confirm co-segregation. The absence of the TT genotype among JAK2V617F-positive patients, compared with its occurrence in 15.2% of JAK2V617F-negative patients, may indicate that the T allele at rs10974900 is less frequently associated with JAK2V617F-positive disease. However, this observation should be interpreted with caution due to the limited number of JAK2V617F-positive cases in the cohort (n = 17).

After stratifying the results by MPN subtype, ET accounted for the highest proportion of haplotype carriers (rs12343867: 56.3% wereTT homozygous). This is consistent with the large PT-1 cohort study by Jones et al. (2010), in which the haplotype was found to predispose specifically to MPL-mutated as well as V617F-positive ET (31). Pardanani et al. further confirmed that the 46/1 haplotype confers ET susceptibility regardless of JAK2V617F status (32). The high 46/1 carrier frequency in ET patients in the present study (80.7% when considering both TT and CT genotypes at rs12343867) far exceeds the 41% C-allele frequency (equivalent to CC+CT combined) reported in literature (32), although direct comparison is complicated by the difference in allele assignment conventions and population demographics.

The present findings carry several potential clinical implications. The findings of this study provide insights regarding the genetic basis of MPNs. The high frequency of the JAK2 46/1 haplotype in this Saudi MPN cohort suggests that germline predisposition testing may be relevant in clinical practice. From a public health perspective, the identification of high-risk 46/1 carriers could inform surveillance strategies for family members. The 46/1 haplotype has been associated with several disease-related complications, including splanchnic vein thrombosis, Budd–Chiari syndrome, elevated red blood cell counts, increased platelet and leukocyte counts, and higher hemoglobin levels (5), as well as inflammatory dysregulation that may contribute to disease progression (8). Understanding haplotype status may help assess the risk of thrombotic and hemorrhagic complications, which are the primary causes of morbidity and mortality. Molecular characterization is also essential for identifying suitable patients and initiating targeted therapies like JAK2 inhibitor therapy (33).

To our knowledge, this is the first study in Saudi Arabia to simultaneously characterize both JAK2V617F mutation prevalence and the germline JAK2 46/1 haplotype in a MPN cohort. The use of a validated quantitative PCR approach for mutation detection and TaqMan SNP genotyping assays for haplotype analysis reflects current best practice. A relatively large cohort of 130 clinically diagnosed MPN patients was included, increasing the reliability of the findings. The inclusion of non-MPN controls provides a reference distribution, albeit with acknowledged sample size limitations. The study also represents a clinically important contribution to the effort to understand MPN heterogeneity across global populations, building on prior reports from European, North American, Japanese, Brazilian, and Moroccan cohorts (22–24, 26, 28, 29, 31, 32, 34–36).

This study has several limitations that should be acknowledged. First, the sample size of 130 MPN patients limits subgroup analyses of the four MPN diagnostic categories. The JAK2V617F-positive subgroup is too small for robust statistical comparison with the V617F-negative group. Second, the study does not include healthy age- and sex-matched Saudi controls for haplotype frequency determination. This limits the ability to draw definitive conclusions about whether the observed 46/1 carrier frequency represents an over-representation relative to the healthy Saudi population. Third, the absence of CALR and MPL mutation testing in the present study is a significant limitation. The majority (86.9%) of patients were JAK2V617F-negative, and many are likely to harbor alternative driver mutations whose relationship to the 46/1 haplotype has not been assessed here. Fourth, the study was conducted at a single center, which may limit the generalizability of the findings to other populations.

## 5 Conclusion

In summary, this study presents the first characterization of JAK2V617F mutation and the germline JAK2 46/1 haplotype (rs12343867) in a Saudi Arabian MPN cohort. The relatively low JAK2V617F positivity rate likely reflects the predominance of ET in the cohort and the high burden of JAK2-negative disease requiring further molecular characterization. The high 46/1 haplotype carrier frequency is consistent with published data from other global populations and supports its role as a germline predisposition factor relevant to MPN pathogenesis in Saudi patients. These findings propose the JAK2 46/1 haplotype as a candidate supplementary biomarker for MPN risk stratification in Saudi Arabia. Prospective, multicentre studies with larger patient cohorts, driver mutation screening (including CALR and MPL), healthy control comparison, and allele burden quantification are required for a more elaborate understanding.

## Data Availability

The datasets generated and/or analyzed during the current study are not publicly available due to ethical and privacy considerations related to human genetic and clinical data. De-identified data may be made available upon reasonable request, subject to applicable institutional and ethical approvals. Requests should be directed to the corresponding author, Sana Alqarni (saalqarni@ksu.edu.sa).
