# Supplementary material for: Characterization of JAK2V617F and the JAK2 46/1 germline haplotype in myeloproliferative neoplasms in a Saudi Arabian cohort: a case-based analysis
Source: Front Oncol. 2026 Sep 15;16:1910710. doi: 10.3389/fonc.2026.1910710 (PMC13619491; doi:10.3389/fonc.2026.1910710)
Supplement: Supplementary file 1 [file Table1.docx]

**Supplementary Materials**

Supplementary Table 1. Primer sequences used for the detection of JAK2 V617F mutation and genotyping of JAK2 46/1 haplotype SNPs

| Gene/SNP ID | Primer Type | Sequence (5′–3′) |
| --- | --- | --- |
| JAK2V617F | Forward primer | 5o- ACAACAGTCAAACAACAATTC -3C |
|  | Reverse primer | 5e- ACACCTAGCTGTGATCC -3′ |
| rs 12343867 | Forward primer | 5′- GTGTTCATGATAGCAAAAGAGATTATG -3′ |
|  | Reverse primer | 5′- TAGCCAAAAAATATATCTAGTATCATATCATCA -3′ |
| rs 10974900 | Forward primer | 5′- AAATGAAAGAACTGGGCAGA -3′ |
|  | Reverse primer | 5′- CTGCCTGTAACTCGTGTTTCCTCC -3′ |

Supplementary Table 2. Thermal cycling conditions for JAK2 V617F real-time PCR on the Rotor-Gene Q platform

| Step | Temperature (°C) | Duration | Cycles |
| --- | --- | --- | --- |
| Hold 1 (UNG activation) | 50 | 2 min | 1 |
| Hold 2 (Polymerase activation) | 95 | 10 min | 1 |
| Denaturation | 92 | 15 sec | 50 |
| Annealing/Extension | 60 | 1 min | 50 |

Supplementary Table 3: Percentile age groups tested

| **Age Group (years)** | **n** | **% of Cohort** | **Cumulative %** |
| --- | --- | --- | --- |
| <10 | 5 | 3.8 | 3.8 |
| 10–<20 | 11 | 8.5 | 12.3 |
| 20–<30 | 19 | 14.6 | 26.9 |
| 30–<40 | 27 | 20.8 | 47.7 |
| 40–<50 | 26 | 20.0 | 67.7 |
| 50–<60 | 15 | 11.5 | 79.2 |
| ≥60 | 27 | 20.8 | 100.0 |
